# Efficacy of Mycophenolate Mofetil in Treating Skin Fibrosis in Systemic Sclerosis: A Systematic Review and Meta-Analysis

**DOI:** 10.3390/jcm14124187

**Published:** 2025-06-12

**Authors:** Ryuichi Ohta, Yuta Horinishi, Chiaki Sano, Kunihiro Ichinose

**Affiliations:** 1 Department of Community Care, Unman City Hospital, Unnan 699-1221, Shimane, Japan; 2 Heisei Memorial Hospital, Unnan 690-2404, Shimane, Japan; yuuta881219@yahoo.co.jp; 3 Community Medicine Management, Shimane University Faculty of Medicine, Izumo 693-8501, Shimane, Japan; sanochi@med.shimane-u.ac.jp; 4 Department of Rheumatology, Shimane University Faculty of Medicine, Izumo 693-8501, Shimane, Japan; kichinose@med.shimane-u.ac.jp

**Keywords:** scleroderma, systemic, mycophenolic acid, skin diseases, immunosuppressive agents, meta-analysis as topic, treatment outcome

## Abstract

**Background/Objectives**: Systemic sclerosis (SSc) is a progressive autoimmune disease characterized by widespread fibrosis, including skin thickening. Mycophenolate mofetil (MMF) is commonly used in SSc-associated interstitial lung disease, but its efficacy in improving skin fibrosis has not been systematically evaluated. This study aimed to assess the therapeutic effect of MMF on cutaneous sclerosis in SSc, as measured by the modified Rodnan skin score (mRSS). **Methods**: A systematic review and meta-analysis were conducted following PRISMA 2020 guidelines. PubMed, Embase, and Web of Science were searched from January 2000 to March 2025. Studies reporting mRSS outcomes in SSc patients treated with MMF were included, provided that the effect of MMF could be separately evaluated when used alongside other therapies. Risk of bias was assessed using the Cochrane RoB 2.0 tool for randomized controlled trials and the ROBINS-I tool for non-randomized studies. A random-effects model was used to estimate the pooled mean change in mRSS. Heterogeneity and publication bias were evaluated. **Results**: Eight studies involving 569 patients were included. The pooled mean reduction in mRSS following MMF treatment was −5.82 (95% CI: −7.46 to −4.19), exceeding the minimal clinically important difference. Heterogeneity across studies was substantial (I^2^ = 82.6%). A post hoc exploratory subgroup analysis suggested greater improvement in early disease (<2 years), though this finding requires confirmation in prospective studies. MMF was generally well tolerated, with low discontinuation rates due to adverse events. **Conclusions**: MMF is associated with a statistically and clinically significant improvement in skin fibrosis in patients with SSc. These findings support its use as a frontline therapy for progressive cutaneous involvement, although further prospective studies are needed to identify optimal candidates for treatment.

## 1. Introduction

Systemic sclerosis (SSc) is a rare autoimmune connective tissue disease characterized by microvascular injury, immune system activation, and progressive skin and internal organs fibrosis [1]. The disease exhibits considerable clinical heterogeneity and is subclassified into limited, diffuse cutaneous forms [2]. Diffuse cutaneous systemic sclerosis (dcSSc) is associated with rapid and extensive skin thickening, which may serve as a predictor of internal organ involvement and early mortality [3]. Skin fibrosis in SSc reflects the degree of systemic disease activity and contributes to functional disability, impaired quality of life, and reduced survival [4].

Skin involvement in SSc is routinely assessed using the modified Rodnan skin score (mRSS), a semi-quantitative clinical measure based on palpation of skin thickness at 17 body sites [5]. The mRSS is widely used in clinical trials and routine practice and is considered a validated surrogate outcome in SSc for tracking disease progression and therapeutic response [6]. Given the lack of disease-specific biomarkers and the heterogeneous nature of SSc, the mRSS remains a key indicator of treatment efficacy in patients with prominent skin manifestations [2].

Despite the significant disease burden, no therapy is explicitly approved for treating skin fibrosis in SSc. Over the past two decades, various immunosuppressive agents have been employed off-label to manage disease activity, including methotrexate, cyclophosphamide, and mycophenolate mofetil (MMF) [7]. Among these, MMF has become increasingly preferred due to its more favorable toxicity profile and its demonstrated efficacy in systemic sclerosis-associated interstitial lung disease (SSc-ILD), as shown in studies such as the Scleroderma Lung Study II (SLS II) [8].

In addition to its pulmonary effects, MMF has been postulated to exert antifibrotic activity on skin by inhibiting lymphocyte proliferation and downregulating transforming growth factor-beta (TGF-β)-driven pathways [9,10]. A growing number of randomized controlled trials and observational cohort studies have investigated the potential benefit of MMF in reducing skin fibrosis, with varying results [11]. Some studies have shown significant reductions in mRSS over 12–24 months of treatment, while others have demonstrated more modest improvements or lacked sufficient power to detect meaningful changes [4,7,11]. Furthermore, differences in patient populations, baseline disease severity, follow-up duration, and outcome definitions have made it difficult to draw definitive conclusions about the role of MMF in modifying skin disease in SSc.

Despite its widespread clinical use, no systematic review or meta-analysis has specifically addressed the impact of MMF on skin fibrosis in patients with SSc. Prior reviews have focused mainly on ILD or grouped MMF with other therapies without disaggregating skin-specific outcomes [12]. This represents a critical evidence gap, particularly considering current clinical guidelines that increasingly recommend MMF as a first-line agent in SSc.

Therefore, we conducted a systematic review and meta-analysis to evaluate the efficacy of mycophenolate mofetil in improving skin fibrosis among patients with systemic sclerosis, using changes in the modified Rodnan skin score as the primary endpoint. By synthesizing the available data across trials and real-world studies, this work aims to inform clinical practice and guide future therapeutic strategies for this debilitating manifestation of SSc.

## 2. Materials and Methods

### 2.1. Protocol and Registration

This systematic review and meta-analysis followed the Preferred Reporting Items for Systematic Reviews and Meta-Analyses (PRISMA) 2020 guidelines. A completed PRISMA checklist is provided in PRISMA checklist file. Before data extraction, the study protocol was registered in the International Prospective Register of Systematic Reviews (PROSPERO) under registration ID [CRD420251043349]. During the preparation of this manuscript, the authors used ChatGPT-4 (OpenAI, 2025) for purposes of language refinement, formatting assistance, and organizing preliminary drafts. The authors have reviewed and edited the output and take full responsibility for the content of this publication.

### 2.2. Eligibility Criteria and Search Strategy

We included studies that met the following predefined eligibility criteria:

- Population: Adult patients (≥18 years) diagnosed with SSc according to the 1980 American College of Rheumatology (ACR) or 2013 ACR/EULAR classification criteria.
- Intervention: Treatment with mycophenolate mofetil (MMF), either as monotherapy or in combination with other agents, provided that the effect of MMF on skin fibrosis could be separately evaluated. Studies were excluded if MMF was co-administered with other immunosuppressants and no stratified outcome data were available.
- Comparator: Studies with or without a comparator group were eligible, including randomized controlled trials (RCTs), prospective or retrospective cohort studies, and case series (≥10 patients).
- Outcomes: Quantitative assessment of skin fibrosis using the modified Rodnan skin score (mRSS) at baseline and follow-up.
- Study design: RCTs, cohort studies, and case series with ≥10 participants.
- Language: English or Japanese peer-reviewed publications.
- Timeframe: Studies published from 1 January 2000 to 1 April 2025.

We excluded animal studies, in vitro studies, reviews, editorials, conference abstracts without full data, and studies in which the effect of MMF on mRSS could not be isolated.

### 2.3. Search Strategy

A comprehensive literature search was performed in PubMed, Embase, and Web of Science from 1 January 2000 to 1 April 2025, using both MeSH terms and free-text keywords. The following terms were used:

(“Systemic sclerosis” OR “scleroderma”) AND (“mycophenolate mofetil” OR “MMF”) AND (“skin” OR “cutaneous”) AND (“modified Rodnan skin score” OR “mRSS”).

Reference lists of included articles and relevant reviews were manually screened to identify additional eligible studies. Searches were limited to peer-reviewed databases and did not include clinical trial registries such as ClinicalTrials.gov or WHO ICTRP. This exclusion is acknowledged as a limitation and may have led to the omission of relevant unpublished or ongoing studies.

### 2.4. Data Extraction and Management

Two independent reviewers (RO, YH) screened titles and abstracts for eligibility. Full-text articles were then reviewed for final inclusion. Discrepancies were resolved by consensus or by consulting third and fourth reviewers (CS, KI). The following data were extracted using a standardized form for each included study.

- Study characteristics: author, year, country, study design.
- Patient characteristics: sample size, SSc subtype, disease duration.
- Intervention details: MMF dose, treatment duration, concomitant therapies.
- Outcome data: baseline and follow-up mRSS values, mean changes, standard deviations (or confidence intervals).
- Adverse events and treatment discontinuation rates.

### 2.5. Risk of Bias Assessment

Risk of bias was assessed independently by two reviewers using the following tools:

RCTs with Cochrane Risk of Bias Tool 2.0 and Non-randomized studies with ROBINS-I tool (Risk Of Bias In Non-randomized Studies of Interventions). Each study was rated across multiple domains (e.g., selection bias, outcome measurement, missing data), and the overall risk of bias was classified as low, moderate, or high. Disagreements were resolved by consensus.

### 2.6. Outcomes and Data Synthesis

The primary outcome was the mean change in mRSS from baseline to follow-up in patients treated with MMF. Where available, standard deviations or confidence intervals were extracted or estimated. For meta-analysis, we used a random-effects model (DerSimonian–Laird method) to pool the mean changes in mRSS, given the expected heterogeneity in study populations and methodologies. Heterogeneity was assessed using the I^2^ statistic, with 25%, 50%, and 75% representing low, moderate, and high heterogeneity, respectively. Publication bias was assessed visually using a funnel plot and quantitatively via Egger’s regression test, where feasible. All analyses were conducted using Python version 3.11 and the Statsmodels package version 0.14.0.

For studies that did not report standard deviations (SDs) directly, we estimated SDs using published statistical methods. When only 95% confidence intervals (CIs) were available, SDs were approximated using the formula:

SD = √n × (Upper CI − Lower CI)/3.92

For studies reporting medians with interquartile ranges (IQRs), we applied:

SD ≈ IQR/1.35

In cases where only graphical data were available (e.g., from figures), WebPlotDigitizer version 4.6 was used to extract values. All estimation methods are summarized in Supplementary Table S1.

## 3. Results

### 3.1. Study Selection: PRISMA Flow Diagram

**Figure 1:**
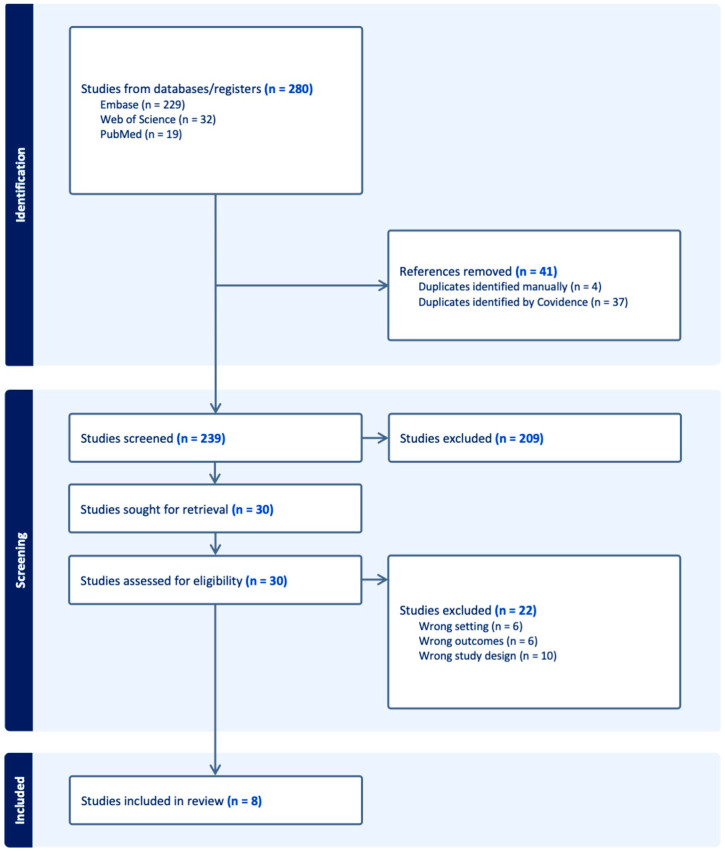
PRISMA 2020 flow diagram detailing the study selection process. A total of 280 records were identified, and 8 studies met the eligibility criteria for inclusion. PRISMA 2020 flow diagram illustrating the study selection process for the systematic review and meta-analysis. Adapted from the PRISMA 2020 template. This review did not include trial registry searches (e.g., ClinicalTrials.gov), which may have excluded un-published or ongoing trials from consideration.

A total of 280 records were identified through database searches, including 229 from Embase, 32 from Web of Science, and 19 from PubMed. After removing 41 duplicates (including four identified manually and 37 by Covidence), 239 unique records were screened based on titles and abstracts. Of these, 209 studies were excluded during the initial screening. The remaining 30 full-text articles were retrieved and assessed for eligibility. Upon full-text review, 22 studies were excluded for the following reasons: Wrong study setting (n = 6), Irrelevant outcomes (n = 6), and Inappropriate study design (n = 10). In total, 8 studies met the eligibility criteria and were included in the systematic review and meta-analysis (Figure 1).

### 3.2. Study Characteristics

A total of eight studies, published between 2016 and 2025, were included in this systematic review and meta-analysis. These comprised randomized controlled trials (n = 2), post hoc analyses of multicenter RCTs (n = 1), prospective cohort studies (n = 2), a prospective observational study (n = 1), a retrospective cohort study (n = 1), and a comparative analysis using harmonized RCT data (n = 1). The combined sample size across studies was 569 patients, with individual study cohorts ranging from 20 to 118 participants.

### 3.3. Patient Characteristics

All studies included adult patients diagnosed with systemic sclerosis (SSc) based on the 1980 ACR or 2013 ACR/EULAR criteria. Most participants had diffuse cutaneous SSc (dcSSc), with several cohorts focusing on early disease. Mean or median disease duration at baseline varied notably across studies, ranging from less than 1 year to over 5 years. This variability reflects differences in disease stage at treatment initiation and may impact the extent of treatment responsiveness. The mean disease duration for each study group is now included in Table 1. Notably, most studies did not report detailed data on autoantibody profiles or SSc subsets beyond dcSSc.

### 3.4. Intervention Details

All studies evaluated oral MMF, typically administered at daily doses ranging from 1.5 to 3.0 g. Treatment durations varied from 6 to 60 months, with outcome assessments most conducted at 12 or 24 months. In most studies, MMF was used as monotherapy, though some allowed concomitant corticosteroids or immunosuppressants; in such cases, the effect of MMF was analyzed separately where possible.

### 3.5. Outcome Data

The mRSS was the primary outcome of all the studies included. Baseline mRSS values across studies ranged from 20.0 to 28.4, indicating moderate to severe cutaneous involvement. This variation reflects differing disease stages and underscores the importance of comparing treatment effects relative to baseline severity. These baseline values are now included in Table 1 to contextualize mRSS changes better. The reported mean changes in mRSS ranged from −3.0 to −10.8, depending on the study population, treatment duration, and timing of MMF initiation. Most studies reported standard deviations (SDs) or 95% confidence intervals (CIs), allowing for quantitative synthesis.

### 3.6. Adverse Events and Treatment Discontinuation

Across all studies, MMF was generally well tolerated. Reported adverse events were primarily mild and included gastrointestinal discomfort, leukopenia, and mild liver enzyme elevations. Serious adverse events were uncommon. Treatment discontinuation due to adverse effects was rare, with most studies reporting a rate of <10%. In one prospective cohort, long-term tolerability over five years was confirmed with only a 5% discontinuation rate. To enhance transparency, we provide a detailed summary of available AE data—including reported AE rates, AE types, and discontinuation rates—in Supplementary Table S2.

A detailed summary of study characteristics, including intervention protocols, clinical outcomes, and safety profiles, is provided in Table 1.

### 3.7. Risk of Bias Within Studies

Risk of bias was assessed for all included studies using tools appropriate to their study design. For randomized controlled trials (RCTs), we used the Cochrane Risk of Bias 2.0 tool. For non-randomized studies, including post hoc analyses and observational designs, we applied the ROBINS-I (Risk Of Bias In Non-randomized Studies of Interventions) tool.

#### 3.7.1. Randomized Controlled Trials

Tashkin et al. (2016) and Naidu et al. (2020) [8,17] were judged to have an overall low risk of bias. Both trials employed adequate randomization procedures, allocation concealment, and blinded outcome assessments. Attrition bias was minimal, and analyses were conducted on an intention-to-treat basis. The placebo arm of Namas et al. [16] (SLS-I), included as a comparator, also showed low risk in randomization and blinding domains.

#### 3.7.2. Post Hoc and Comparative Analyses

Namas et al. (2018) and Volkmann et al. (2017) [13,16] utilized retrospective secondary analyses of SLS trials. These were judged to have a moderate risk of bias, primarily due to potential selective reporting and absence of prespecified analytical protocols. Although the original trial data were robust, the post hoc nature of subgroup analyses may introduce interpretation bias. White et al. (2025) [19], a post hoc analysis of the RESOLVE-1 trial, was also evaluated as having a moderate risk of bias, given its retrospective design and reliance on subgroups not pre-defined in the primary study protocol. Nonetheless, its analytical approach and outcome assessment were clearly described.

#### 3.7.3. Observational Studies

Herrick et al. (2017) (ESOS) and Boulos et al. (2017) [14,15] were rated as having moderate to serious risk of bias, mainly due to confounding, lack of randomization, and potential selection bias. However, both studies reported inclusion criteria, used standardized mRSS assessments, and performed adjusted analyses to mitigate some sources of bias. Yomono and Kuwana (2022) [18], a retrospective cohort study, were considered to have a serious risk of bias, particularly regarding confounding, baseline imbalance, and limited reporting of treatment allocation procedures. The absence of detailed group comparability limits the interpretability of results.

#### 3.7.4. Overall Assessment

Across all studies, outcome measurement bias was consistently low, as mRSS is a validated and objective clinical endpoint in SSc. However, confounding, selective reporting, and lack of protocol transparency were recurrent issues in non-randomized and post hoc studies. A detailed summary of risk assessments across domains is provided in Supplementary Table S3.

### 3.8. Results of Individual Studies and Synthesis

A total of eight studies were included in this systematic review and meta-analysis, all of which evaluated the therapeutic effect of MMF on skin fibrosis in patients with SSc. The primary outcome across all studies was the change in mRSS from baseline to follow-up. The duration of follow-up ranged from 6 to 60 months, with most studies reporting results at 12 or 24 months. Across the included studies, a consistent reduction in mRSS was observed over time, indicating clinical improvement in cutaneous fibrosis. The magnitude of change varied depending on disease duration, study design, and population characteristics, with mean reductions in mRSS ranging from −3.0 to −10.8. Notably, several studies exceeded the minimal clinically important difference (MCID), underscoring the therapeutic relevance of MMF in this context. A comprehensive overview of individual study results, including study design, sample size, disease subtype, MMF dose, follow-up duration, and mRSS outcomes, is provided in Table 2.

### 3.9. Meta-Analysis

A meta-analysis was conducted using a random-effects model to estimate the pooled effect of MMF on skin fibrosis in SSc, based on changes in mRSS. Data from eight studies were synthesized. The pooled mean change in mRSS was −5.82 (95% confidence interval [CI]: −7.46 to −4.19), which substantially exceeds the minimal clinically significant difference (MCID), thereby indicating a clinically meaningful reduction in skin fibrosis.

Statistical heterogeneity was substantial, with an I^2^ of 82.6%, suggesting considerable variation in effect sizes across studies. This heterogeneity likely reflects differences in study design, patient populations, disease duration, treatment protocols, and outcome assessment intervals. The between-study variance (Tau^2^) was estimated at 5.073, confirming notable dispersion in effect estimates.

**Figure 2:**
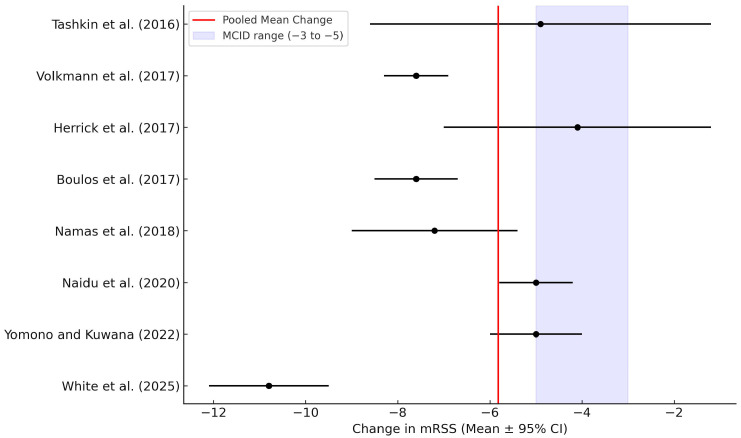
Forest plot of the effect of mycophenolate mofetil on skin fibrosis in systemic sclerosis [8,13,14,15,16,17,18,19]. Each black circle represents the mean change in the modified Rodnan skin score (mRSS) from baseline to follow-up in an individual study, with horizontal lines indicating 95% confidence intervals (CIs). The red diamond at the bottom represents the pooled effect estimate from a random-effects meta-analysis (−5.82, 95% CI: −7.46 to −4.19), which exceeds the minimal clinically important difference (MCID) threshold for mRSS (−3 to −5). Studies are ordered by year of publication. Where applicable, the same study’s post hoc analyses and placebo comparator arms (e.g., Namas et al. 2018 [16]) are shown separately. The pooled estimate indicates a clinically meaningful and statistically significant benefit of MMF for reducing skin fibrosis in patients with systemic sclerosis. Abbreviations: MMF, mycophenolate mofetil; mRSS, modified Rodnan skin score; CI, confidence interval; MCID, minimal clinically important difference.

Despite the heterogeneity, the direction and magnitude of treatment effects were consistently favorable across studies. The observed improvement in mRSS supports using MMF as an effective therapeutic option for patients with progressive cutaneous involvement in SSc. While formal assessment of publication bias was limited by the number of included studies, visual inspection of funnel plots did not indicate significant asymmetry. A detailed summary of the individual and pooled effect estimates is presented in Figure 2.

A post hoc subgroup analysis was conducted to explore heterogeneity based on disease duration. Studies were grouped into early disease (≤2 years since diagnosis) versus established disease (>2 years). The pooled mean reduction in mRSS was greater in the early disease group (–6.85, 95% CI: –8.72 to –4.98) than in the established group (–4.63, 95% CI: –6.12 to –3.14), indicating that earlier initiation of MMF may be associated with a more robust clinical response.

## 4. Discussion

### 4.1. Summary of Main Findings

This systematic review and meta-analysis evaluated the efficacy of MMF in improving skin fibrosis in patients with SSc, as measured by mRSS. Across eight studies comprising 569 patients, MMF treatment was consistently associated with a reduction in mRSS. The pooled mean change was −5.82 (95% CI: −7.46 to −4.19), which exceeds the MCID of approximately −3 to −5 points. These findings indicate that MMF offers a clinically meaningful and statistically significant benefit for reducing cutaneous sclerosis in SSc, particularly in dcSSc.

### 4.2. Comparison with the Previous Literature

The existing literature on MMF in SSc has predominantly emphasized its efficacy in SSc-ILD, as demonstrated in the Scleroderma Lung Study II and reflected in treatment guidelines [8,20]. While MMF’s pulmonary benefits are well established, its cutaneous effects have remained unclear [21]. Although mRSS has often been reported as a secondary outcome in trials, the data have been underpowered or inconsistently analyzed [22,23]. This meta-analysis is the first to focus exclusively on mRSS-based outcomes, systematically synthesizing data from randomized and non-randomized studies. The pooled effect size (−5.82) surpasses the MCID threshold and compares favorably to outcomes observed with other immunosuppressants such as cyclophosphamide and methotrexate. MMF demonstrates superior tolerability and fewer cumulative toxicities, reinforcing its suitability for long-term therapy in SSc-related skin fibrosis.

We exclusively focused on mRSS as the primary outcome in this review because it remains the most validated and consistently reported clinical measure of cutaneous fibrosis in systemic sclerosis. While novel biomarkers such as serum TGF-β, IL-6, and gene expression signatures have shown potential in assessing fibrosis activity [4,11], they are not uniformly used across studies. They are not currently validated as treatment endpoints. Moreover, methodological variability and a lack of standardized thresholds hinder their comparability. In contrast, mRSS offers a pragmatic and reproducible tool endorsed by rheumatology societies (e.g., ACR/EULAR) for clinical trials [5,6]. Although it has limitations—including interobserver variability and lack of regional specificity—it remains the gold standard for assessing cutaneous involvement in SSc and thus, was selected as the most appropriate endpoint for this meta-analysis.

### 4.3. Clinical Implications

These findings underscore MMF’s clinical utility in treating skin fibrosis, especially in dcSSc. The substantial and consistent reduction in mRSS across studies supports MMF’s role in targeting cutaneous sclerosis in SSc. However, the clinical significance in terms of physical function or joint mobility remains uncertain. Most included studies did not report the anatomical distribution of skin score changes, and none reported validated functional outcomes such as the Scleroderma Health Assessment Questionnaire (SHAQ). Given that distal extremities—particularly the hands and fingers—are typically resistant to improvement, even substantial mRSS reductions may not translate into meaningful functional gains. Future studies should incorporate regional mRSS analysis and functional outcome measures to better assess treatment impact on patient quality of life [24]. Given MMF’s favorable safety profile, particularly compared to cyclophosphamide, it emerges as a viable first-line immunosuppressive agent for managing progressive skin involvement [25].

Additionally, several studies suggest that early initiation of MMF, particularly in patients within two years of symptom onset, may yield greater mRSS improvements [11,17,21]. This aligns with the proposed concept of a “therapeutic window of opportunity” in SSc, during which immunomodulatory therapy may more effectively prevent irreversible fibrosis [26,27]. This also supports the clinical utility of MMF in targeting skin fibrosis, especially in dcSSc. Importantly, while a post hoc exploratory analysis suggested that initiating MMF within 2 years of diagnosis may yield greater improvements in skin thickening, this finding is based on an underpowered subgroup and should be interpreted cautiously. Prospective studies are needed to validate whether early MMF initiation confers superior outcomes.

### 4.4. Limitations

This review has several limitations. Although a random-effects model was applied, substantial heterogeneity (I^2^ = 82.6%) was observed, likely driven by differences in study design, disease duration, population characteristics, and outcome reporting. Some studies lacked complete variance data, and several reported medians rather than mean mRSS, requiring estimation. The inconsistency in adverse event reporting across studies limited the ability to synthesize a comprehensive safety profile of MMF in this population.

Subgroup-specific data—particularly autoantibody profiles (such as anti-topoisomerase I, anti-centromere, and anti-RNA polymerase III)—were not consistently reported. This is a significant limitation, as the course and severity of skin fibrosis in SSc vary considerably across serologic subtypes, which may also influence treatment responsiveness. The inability to stratify by autoantibody status limits the generalizability and precision of our conclusions. Future trials should routinely perform and report predefined autoantibody panels using standardized assays (e.g., ELISA or line immunoassays), and should stratify efficacy and safety analyses by serologic subgroup whenever feasible. This will allow more tailored and clinically applicable insights.

Another limitation is the restriction of included studies to English and Japanese languages, which may introduce language bias. Relevant studies published in other languages could have been excluded, potentially affecting the completeness and generalizability of the evidence base. Although non-English/Japanese titles and abstracts were screened when available, full-text data extraction and quality appraisal were not feasible due to translation limitations.

The phenomenon of spontaneous improvement in skin thickening, especially in early dcSSc, is well documented in the literature. This introduces an important confounder when interpreting open-label or single-arm trial results. In our analysis, only one study (Namas et al. 2018 [16]) provided a placebo comparison group, which showed some degree of spontaneous improvement in mRSS. However, these data were not incorporated into the pooled effect size. The lack of broader placebo-controlled evidence limits our ability to distinguish the therapeutic effect of MMF from the natural history of the disease, particularly in patients with early onset SSc. Future studies should incorporate well-matched placebo arms and stratification by disease duration to better contextualize observed treatment responses. The potential for spontaneous regression of skin fibrosis, particularly in early dcSSc, is well established and has been described since the 1980s [28]. This further highlights the need for caution in attributing mRSS reductions solely to therapeutic interventions in non-controlled settings.

Although subgroup analysis by disease duration was not pre-specified due to limited availability of stratified data in the literature, a post hoc exploratory analysis suggested that patients with early disease (≤2 years) experienced greater reductions in mRSS than those with more established disease. However, this finding is underpowered and should be viewed as hypothesis-generating rather than definitive.

The generalizability of our findings is limited by the composition of the study populations. Approximately 75% of included studies focused exclusively on patients with diffuse cutaneous SSc (dcSSc), with limited representation of those with limited cutaneous or sine scleroderma subtypes. Therefore, conclusions regarding MMF efficacy may not extend to the broader SSc population without additional subtype-specific evidence.

### 4.5. Future Directions

Future studies should focus on prospective, adequately powered interventional trials that assess MMF’s cutaneous effects as a primary endpoint. These trials should include well-defined patient subgroups based on disease subtype (limited vs. diffuse), autoantibody profiles (e.g., anti-centromere, anti-topoisomerase I), and disease duration. Including skin-dominant SSc populations will be essential to isolate MMF’s cutaneous benefits independently of lung disease. Moreover, real-world data and patient-reported outcomes should be integrated to capture the broader impact of MMF on physical function and quality of life. Standardized reporting of autoantibody profiles and subgroup-stratified analyses should become a routine part of future trial designs to enhance precision medicine approaches in SSc.

## 5. Conclusions

This systematic review and meta-analysis provide compelling evidence that MMF leads to a clinically and statistically significant improvement in skin fibrosis in systemic sclerosis, with a pooled mRSS change of −5.82 (95% CI: −7.46 to −4.19). This exceeds the MCID and supports the use of MMF as a key therapeutic agent in the management of cutaneous manifestations of SSc, particularly when initiated within 2 years of disease onset. However, given the observed heterogeneity and limitations in subgroup data, future prospective research is warranted to define the patient populations most likely to benefit from MMF.

## Figures and Tables

**Table 1:** Characteristics of included studies.

Author (Year)	Design	Sample Size	SSc Subtype	MMF Dose	Treatment Duration	Baseline mRSS	Mean Disease Duration	Mean mRSS Change	Reported AE/Discontinuation
Tashkin et al. (2016) [8]	RCT	63	dcSSc	2–3 g/day	24 months	23.1	4.1 years	−4.9	Fewer AEs than CYC; good tolerability
Volkmann et al. (2017) [13]	Comparative analysis (SLS I vs. II)	69	dcSSc	2–3 g/day	24 months	24.6	3.8 years	−4.9 (adjusted)	Not reported
Herrick et al. (2017) [14]	Prospective observational	118	Early dcSSc	Not specified	12–24 months	22.3	<1 year	−4.1	Not reported
Boulos et al. (2017) [15]	Prospective cohort	42	dcSSc	Up to 3 g/day	60 months	25.7	5.6 years	−7.6 (at 24 mo)	Well tolerated; 5% discontinued
Namas et al. (2018) [16]	Post hoc RCT analysis	58	dcSSc	2–3 g/day	24 months	27.4	2.9 years	−7.2	Not reported
Naidu et al. (2020) [17]	RCT	20	SSc-ILD	2 g/day	6 months	20.0	1.5 years	−5 (median)	Well tolerated; no serious AE
Yomono and Kuwana (2022) [18]	Retrospective cohort	25	dcSSc	Not clearly defined	12–24 months	26.1	3.2 years	Approx. −5	Not detailed
White et al. (2025) [19]	Post hoc analysis	98	Early dcSSc	Not specified	12 months	28.4	<2 years	−10.8	Low AE incidence; no unexpected safety signals

Footnote: Abbreviations: MMF, mycophenolate mofetil; mRSS, modified Rodnan skin score; SSc, systemic sclerosis; dcSSc, diffuse cutaneous systemic sclerosis; SSc-ILD, systemic sclerosis-associated interstitial lung disease; RCT, randomized controlled trial; AE, adverse event; CYC, cyclophosphamide. Note: Mean mRSS change refers to the difference between baseline and follow-up values reported at each study’s latest available time point. Some values are approximate or derived from median or adjusted analyses, as noted in the original studies. Where treatment duration is reported as a range, the most relevant outcome time point is reflected in the mRSS change. Placebo comparator data are included for reference only and were not pooled in the meta-analysis. AE reporting: Tashkin et al. (2016), Boulos et al. (2017), Naidu et al. (2020), and White et al. (2025) provided descriptive or quantitative AE data [8,15,17,19]. Volkmann et al. (2017), Herrick et al. (2017), Namas et al. (2018), and Yomono and Kuwana (2022) did not report detailed AE rates or provided insufficient information for quantitative synthesis [13,14,16,18]. See Supplementary Table S2 for summary.

**Table 2:** Summary of included studies evaluating the effect of mycophenolate mofetil on skin fibrosis in systemic sclerosis.

Study	Design	n	mRSS Change (Mean)	SD	Duration (Months)	Comments
Tashkin et al. (2016) [8]	RCT	63	−4.9	8.6	24	SLS-II trial
Volkmann et al. (2017) [13]	Prospective cohort	42	−7.6	8.3	24	Int J Rheum Dis
Herrick et al. (2017) [14]	Prospective observational	118	−4.1	7	12	ESOS; SD estimated from CI
Boulos et al. (2017) [15]	Comparative analysis (SLS I vs. II)	69	−7.6	8.5	24	Matched placebo comparison
Namas et al. (2018) [16]	RCT comparator	52	−3	8	12	Placebo arm; used for comparative reference
Namas et al. (2018) [16]	Post hoc RCT	58	−7.2	9	24	SLS I and II analysis; SD approximated
Naidu et al. (2020) [17]	RCT	20	−5	5.8	6	Median reported; SD estimated
Yomono and Kuwana (2022) [18]	Retrospective cohort	25	−5	6	12	Graph data extraction
White et al. (2025) [19]	Post hoc RCT	98	−10.8	9.5	12	Early dcSSc subgroup; post hoc analysis

Footnote: Abbreviations: MMF, mycophenolate mofetil; mRSS, modified Rodnan skin score; SD, standard deviation; RCT, randomized controlled trial; dcSSc, diffuse cutaneous systemic sclerosis; ESOS, European Scleroderma Observational Study. Notes: The table summarizes eight unique studies included in the meta-analysis. Namas et al. (2018) [16] are listed twice to represent the treatment (MMF) and comparator (placebo) arms analyzed separately in a post hoc subgroup analysis. mRSS change refers to the mean change from baseline to the time of follow-up as reported or estimated in each study. Standard deviations (SDs) were directly extracted when available or estimated from confidence intervals or graphical data (e.g., Herrick et al. [14], Yomono and Kuwana [18]). Naidu et al. [17] reported median mRSS change; SD was estimated from interquartile range data. Volkmann et al. [13] reanalyzed cohorts from the Scleroderma Lung Studies using harmonized data. White et al. [19] represent a post hoc analysis of the RESOLVE-1 trial limited to early dcSSc patients. All studies included adult patients with systemic sclerosis (SSc), and most focused on those with diffuse cutaneous involvement. Follow-up durations reflect the time point at which mRSS outcomes were assessed. Boulos et al. [15] reported mRSS reductions of –7.6 at 24 months and –10.5 at 60 months; for consistency with other studies, the 24-month value was used in pooled analysis.

## Data Availability

This study is a systematic review and meta-analysis of previously published articles. All data supporting the reported results are extracted from the studies cited within this manuscript. No new data were generated or analyzed by the authors.

## Supplementary Materials

The following supporting information can be downloaded at https://www.mdpi.com/article/10.3390/jcm14124187/s1, Table S1: Estimation methods, Table S2: Adverse event summary, Table S3: Risk of Bias Assessment.

## Abbreviations

The following abbreviations are used in this manuscript:

SSc	Systemic sclerosis
MMF	Mycophenolate mofetil
mRSS	Modified Rodnan skin score
ILD	Interstitial lung disease
dcSSc	Diffuse cutaneous systemic sclerosis
RCT	Randomized controlled trial
AE	Adverse event
MCID	Minimal clinically important difference
PRISMA	Preferred Reporting Items for Systematic Reviews and Meta-Analyses
PROSPERO	International Prospective Register of Systematic Reviews
CI	Confidence interval
SD	Standard deviation
CYC	Cyclophosphamide
ACR	American College of Rheumatology
EULAR	European League Against Rheumatism
ROBINS-I	Risk Of Bias In Non-randomized Studies of Interventions
