# Supplementary material for: Efficacy of Mycophenolate Mofetil in Treating Skin Fibrosis in Systemic Sclerosis: A Systematic Review and Meta-Analysis
Source: J Clin Med. 2025 Jun 12;14(12):4187. doi: 10.3390/jcm14124187 (PMC12194733; doi:10.3390/jcm14124187)
Supplement: Supplementary file 1 [file jcm-14-04187-s001.zip › jcm-3680003-supplementary.pdf]

Supplementary Table 1: Estimation Methods

Study	Reported Statistic	Estimation Method	Sample Size (n)	CI Width	Estimated SD
Herrick et al. (2017)	95% CI	SD = √n × (Upper CI – Lower CI) / 3.92	118	10	27.71
Naidu et al. (2020)	Median (IQR)	SD ≈ IQR / 1.35	20		5.78
Yomono & Kuwana (2022)	Graphical data	WebPlotDigitizer + range approximation	25		6
Namas et al. (2018) – Post hoc	Range	Estimated SD from range assuming normal distribution	58		9
White et al. (2025)	95% CI	SD = √n × (Upper CI – Lower CI) / 3.92	98	11.2	28.28

Supplementary Table 2: Adverse Event Summary

Study	Reported AE Rate	Types of AEs	Discontinuation Rate due to AEs	Notes
Tashkin et al. (2016)	Not quantified	Fewer AEs than CYC; GI symptoms	Not reported	Good tolerability
Volkmann et al. (2017)	Not reported	Not reported	Not reported	—
Herrick et al. (2017)	Not reported	Not reported	Not reported	—
Boulos et al. (2017)	Not quantified	Mild GI symptoms, leukopenia	5%	5-year follow-up
Namas et al. (2018)	Not reported	Not reported	Not reported	—
Naidu et al. (2020)	No serious AEs reported	Mild GI symptoms	0%	Well tolerated
Yomono & Kuwana (2022)	Not detailed	Not detailed	Not reported	Retrospective cohort
White et al. (2025)	Not quantified	Low AE incidence; no unexpected safety signals	Not reported	Post hoc analysis

Supplementary Table 3: Risk of Bias Assessment

Author (Year)	Study Design	Randomization	Deviations from Intended	Missing Outcome Data	Measurement of Outcome	Selection of Reported	Overall Risk of

			Interventions			Result	Bias
Tashkin et al. (2016)	RCT	Low	Low	Low	Low	Low	Low
Volkmann et al. (2017)	Comparative analysis	Low	Moderate	Low	Low	Moderate	Moderate
Herrick et al. (2017)	Prospective observational	Not applicable	Moderate	Low	Low	Moderate	Moderate
Boulos et al. (2017)	Prospective cohort	Not applicable	Moderate	Low	Low	Moderate	Moderate
Namas et al. (2018)	Post hoc RCT analysis	Low	Moderate	Low	Low	Moderate	Moderate
Naidu et al. (2020)	RCT	Low	Low	Low	Low	Low	Low
Yomono & Kuwana (2022)	Retrospective cohort	Not applicable	Serious	Moderate	Low	Serious	Serious
White et al. (2025)	Post hoc RCT	Low	Moderate	Low	Low	Moderate	Moderate
Namas et al. (2018, placebo)	RCT comparator	Low	Low	Low	Low	Low	Low
